# Ultra-Low Loading of Gold on Nickel Foam for Nitrogen Electrochemistry

**DOI:** 10.3390/nano13212850

**Published:** 2023-10-27

**Authors:** Giuseppe Tranchida, Rachela G. Milazzo, Marco Leonardi, Silvia Scalese, Roberta A. Farina, Salvatore Lombardo, Stefania M. S. Privitera

**Affiliations:** 1 Department of Chemical Sciences, University of Catania, Viale Andrea Doria, 6, 95125 Catania, Italy; 2 Institute for Microelectronics and Microsystems, National Research Council (CNR-IMM), Strada VIII, 5, 95121 Catania, Italy

**Keywords:** nitrogen electroreduction, catalysts, gold nanoparticles, ammonia synthesis

## Abstract

Ammonia (NH_3_) is widely used in various fields, and it is also considered a promising carbon free energy carrier, due to its high hydrogen content. The nitrogen reduction reaction (NRR), which converts nitrogen into ammonia by using protons from water as the hydrogen source, is receiving a lot of attention, since effective process optimization would make it possible to overcome the Haber–Bosch method. In this study, we used a solution-based approach to obtain functionalized porous Ni foam substrates with a small amount of gold (<0.1 mg cm^−1^). We investigated several deposition conditions and obtained different morphologies. The electrochemical performance of various catalysts on the hydrogen evolution reaction (HER) and NRR has been characterized. The ammonia production yield was determined by chronoamperometry experiments at several potentials, and the results showed a maximum ammonia yield rate of 20 µg h^−1^ mg_cat_^−1^ and a Faradaic efficiency of 5.22%. This study demonstrates the potential of gold-based catalysts for sustainable ammonia production and highlights the importance of optimizing deposition conditions to improve the selectivity toward HER.

## 1. Introduction

Nitrogen is one of the most abundant elements, and it is present in the environment in a wide variety of chemical forms, including inorganic nitrogen gas (N_2_), ammonia (NH_3_) and ammonium (NH_4_^+^), nitrite (NO_2_^−^), nitrate (NO_3_^−^), nitrous oxide (N_2_O), nitric oxide (NO) and organic nitrogen [1,2,3]. The processes in the global nitrogen cycle transform nitrogen from one form to another. In particular, the biological nitrogen fixation process converts atmospheric dinitrogen gas (N_2_) into ammonia through nitrogenase proteins. This process of nitrogen reduction is essential for life, since fixed inorganic nitrogen compounds are required for the biosynthesis of all organic compounds containing nitrogen, such as amino acids and proteins, and it is crucial for agriculture [4]. Ammonia is also relevant, as a key precursor for the manufacturing of many nitrogen compounds, including dyes, explosives, resins, etc. Currently, ammonia is industrially produced by the Haber–Bosch process from N_2_ and H_2_ feeding gases and an Fe-based catalyst. However, because of the high bonding energy of N≡N (940.95 kJ mol^−1^), it requires a high temperature (350–550 °C) and pressure (150–350 atm) to break the triple bond of nitrogen molecules [5]. This results in a highly polluting process which accounts for 1–2% of global carbon dioxide emissions [6,7]. Consequently, there is a significant need for eco-friendly alternative methods of producing NH_3_, and electrochemical nitrogen reduction using aqueous electrolytes is considered an interesting approach since it can be powered by renewable electricity inputs [8]. This electrochemical approach would enable on-site distributed ammonia production, supplanting centralized manufacturing, and therefore contributing to decarbonization [9]. Moreover, ammonia is also a very promising and versatile energy vector, which could be used to mitigate the intrinsic fluctuations of renewable energy sources. Unlike hydrogen, ammonia can be easily liquified, which has clear advantages for storage and transportation [10,11]. However, the development of the electrochemical nitrogen reduction reaction (NRR) has been hindered by the lack of effective electrocatalysts that can produce NH_3_ with a high yield and faradaic efficiency (FE) [12,13,14,15,16,17].

In general, nanostructured noble metal materials (Au, Ag, Ru, Ir, etc.) [18,19] are very promising in the field of catalysis, since they possess good conductivity and a lot of surface active sites. It has been speculated that their catalytic activity for nitrogen electroreduction originates from the interaction between their d orbitals and the lone pair electrons of N_2_, resulting in a weakening of its triple bond [20]. Among noble metals, gold is a promising candidate for green ammonia generation, and it has been proved that NRR on Au surfaces is an associative mechanism, with the hydrogenation and the breaking of the triple bonds of N atoms occurring simultaneously [5,21,22,23,24]. Indeed, when the N_2_ triple bond is broken, the Au electronic structure allows hydrogenation and offers a lower energy barrier for the formation and stabilization of N_2_H_2_* intermediates on the catalyst surface [25,26].

The catalytic performance can be improved by the use of porous and/or multifaceted Au surfaces, that ensure the improvement of active sites on the catalyst [27]. Usually, mesoporous precious metals are produced through dealloying or through template-based methods or block copolymer micelles [28,29,30], sometimes with poor control over pores size. In addition, the synthetized materials need to be fixed on the substrate by binders, resulting in the degradation of electrocatalytic performance and/or the introduction of false positives in ammonia quantification procedures [31,32].

In this paper we adopted electroless deposition [33,34,35], based on spontaneous galvanic displacement, to cover the porous substrates of Ni foam with a multifaceted layer made of interconnected gold nanoparticles, avoiding the use of any binder or polymer. The effect of the catalyst’s morphology and coverage on the electrochemical synthesis of ammonia is studied. The catalyst’s morphology is found to be relevant in determining its ability to reduce nitrogen.

## 2. Materials and Methods

### 2.1. Gold Electroless Deposition on Nickel Foam

The nickel foam electrodes with a thickness of 1.6 mm with a porosity of 95% and 20 pores per linear cm were purchased from Sigma-Aldrich (Milan, Italy). Before the deposition, each sample (1 × 1 cm^2^) was pretreated in acetone (CH_3_COCH_3_, Carlo Erba Reagents, Milan, Italy) at 60 °C with sonication and then in a 0.01 M HCl (HCl 37%, Carlo Erba Reagents, Milan, Italy) solution at 60 °C for 30 min, to carry off the native oxide. After cleaning, the Ni foam substrate was placed into a solution containing 1 mM KAuCl_4_ (99.995% trace metal, Sigma-Aldrich, Milan, Italy) and 0.01 M HCl, at room temperature and under ambient light. The initial color of the solution was yellow, but gradually shifted from light yellow to colorless, indicating a reduction in the concentration of AuCl_4_^−^ ion species. The spontaneous reaction between AuCl_4_^−^ and the Ni substrate, which allows the deposition of gold on nickel, is (1):

(1) $$2{\mathrm{AuCl}}_{4}^{-}+3{\mathrm{Ni}}^{0}\to 2{\mathrm{Au}}^{0}+3{\mathrm{Ni}}^{2+}+8{\mathrm{Cl}}^{-}$$

As nickel has a much lower electrochemical potential than gold (−0.25 V vs. +1.00 V), the Au deposition on Ni occurs spontaneously [36]. The reaction takes place through the oxidation of the conductive substrate at an open-circuit potential, wherein Au ions are reduced and deposited in a metallic state and Ni^2+^ ions are dissolved in the HCl solution. Some depositions (indicated in the following as sample A and B) were performed by stirring the solution at 600 rpm and by adding isopropyl alcohol (C_3_H_8_O, IPA, Carlo Erba Reagents, Milan, Italy) at a concentration of 5%, since with these conditions it is possible to obtain uniform coverage on both the outer and inner regions of the porous Ni foam [37]. With the aim of studying the effect of time on the deposition, we prepared samples A and B through immersion in the deposition solution for 30 s and 120 s, respectively. To study the effect of prolonged deposition time, another sample (C) was prepared, in the same way as sample B, for 120 s with IPA and stirring (to ensure uniform coverage), and then immersed again for 1800 s without IPA and stirring, to promote diffusion-limited growth. After deposition, all the samples were abundantly rinsed in deionized water and dried in air.

### 2.2. Characterization

The morphology of the catalyst was investigated by scanning electron microscopy (SEM), using a ZEISS FE-SEM SUPRA 35 (Carl Zeiss AG, Jena, Germany) equipped with an Energy Dispersive X-ray (EDX) microanalysis system (X-MAX, 80 mm^2^ by Oxford Instruments, Abingdon, UK).

The electrochemical characterization was performed in a dual chamber H-cell, filled with a 0.1 M Na_2_SO_4_ solution. The cathodic and the anodic compartments were separated by a ZIRFON PERL membrane (AGFA), since it has been shown that it can avoid contaminations and artifacts that occur with the use of a proton exchange membrane [38]. A Pt wire has been employed as a counter electrode (CE) and a saturated calomel electrode (SCE, saturated KCl), as reference.

Cyclic voltammetry (CV) and chronoamperometry (CA) measurements were performed using a Keithley 2600-Source Current Unit. The catalyst activity of the samples was analyzed by linear sweep voltammetry (LSV) in a quasi-steady-state condition, to avoid distortions generated by charging effects, with a scan rate of 0.4 mV s^−1^ [39]. We adopted a potential range between 0 V and −0.55 V vs. RHE. Electrochemical impedance spectroscopy (EIS) measurements were performed using an Ivium Vertex5A (Ivium Technologies B.V., Eindhoven, The Netherlands) potentiostat with a frequency range from 1 Hz to 1 MHz, by applying a 50 mV small signal amplitude and 0 V bias vs. RHE.

The following equation is used for the conversion from SCE to reversible hydrogen electrode (RHE):

E (vs. RHE) = E (vs. SCE) + 0.243 + 0.059 × pH(2)

### 2.3. Electrochemical Ammonia Synthesis

**Figure 1:**
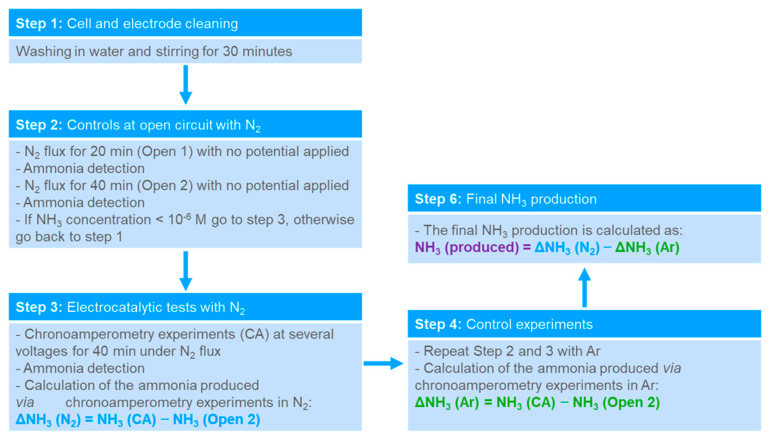
Schematic illustration of the experimental protocol for the electrochemical ammonia synthesis.

Ammonia was produced during chronoamperometry measurements at a constant potential while fluxing N_2_ gas, with a flow rate of 10 mL min^−1^. To avoid external contaminations, a rigorous protocol was adopted [40], as described in Figure 1, including: the washing of the electrodes and cell in water at 35 °C before chronoamperometry measurements; adopting high purity N_2_ and Ar gases (Nippon Gases, Tokyo, Japan, 99.9999% with <0.5 ppm of oxygen); and gas washing in two acidic saturators (10 mM H_2_SO_4_) and water, for further purification before entering the electrochemical cell.

The amount of ammonia in the electrolyte was measured before the experiments (see Figure S7). Then, the cathode and the anode compartments were filled, respectively, with 22 mL and 8 mL of electrolyte, and the amount of ammonia in the solution was measured again after 20 min (Open1) and 40 min (Open2), at open circuit under N_2_ (or Ar) flow, to check the ammonia concentration in the cell. If the concentration of NH_3_ exceeded 10^−6^ M and/or changed over time, the cell was emptied and rinsed again. This method enabled a thorough examination of any internal system contaminations, such as ammonia that was already present in the electrode or in the cell.

The amount of ammonia measured at the open circuit (Open2) was subtracted from the amount measured after the chronoamperometry experiments (CA) with external potential applied, to obtain the net ammonia amount, ΔNH_3_. Ammonia was quantified in both cathodic and anodic compartments but no NH_3_ was detected at the anode. Moreover, an acidic trap was placed at the cathode gas outlet; however, no relevant amount of NH_3_ was detected in this trap after chronoamperometry. As proposed in the literature [41,42], Ar-saturated control experiments were executed at the same bias. In order to eliminate possible contributions from the water other than N_2_, such as nitrates and nitrites, the ammonia produced under an Ar-saturated atmosphere was subtracted from the net amount of ammonia produced in N_2_ at the same conditions, to obtain the Ar-corrected produced NH_3_.

### 2.4. Ammonia Detection

Sodium hydroxide (NaOH, Thermo Fisher Scientific, Monza, Italy), trisodium citrate dihydrate (Na_3_C_6_H_5_O_7_·2H_2_O, Alfa Aesar, Ward Hill, MA, USA, ≥99.0%), salicylic acid (C_7_H_6_O_3_, Alfa Aesar, 99%), sodium pentacyanonitrosylferrate (III) dihydrate (Na_2_[(Fe(CN)_5_NO]·2H_2_O, Alfa Aesar, 98%), ammonium chloride (NH_4_Cl granular, Alfa Aesar, 99.5%) and sodium hypochlorite solution (NaClO, Alfa Aesar, 11–15%) were used to measure the ammonia in the electrolyte using the indophenol blue method, as described by [43,44], with minor adjustments. For this method, 1 mL of the sample was mixed with 1 mL of a solution made up of 1 M NaOH, 5%wt sodium citrate and 5%wt salicylic acid. Then, 0.5 mL of a 0.05 M NaClO solution and 0.3 mL of a 0.1% sodium nitroferricyanide solution were added to the mixture. The solution was incubated in the dark for 30 min to avoid Fe-catalyzed photodegradation. A UV-vis spectrophotometer (Cary 5000 UV-Vis-NIR, Agilent, Santa Clara, CA, USA) was used to measure the solution’s absorbance, at a wavelength of 655 nm.

A calibration curve was plotted, to quantify the amount of NH_3_ produced using standard ammonia chloride solutions ranging from 0.15 to 0.015 μg mL^−1^ in Na_2_SO_4_ electrolyte solution. Figure S1 shows the typical absorption spectra of standard ammonia solutions and the corresponding calibration curve.

## 3. Results and Discussion

### 3.1. Morphological Characterization

**Figure 2:**
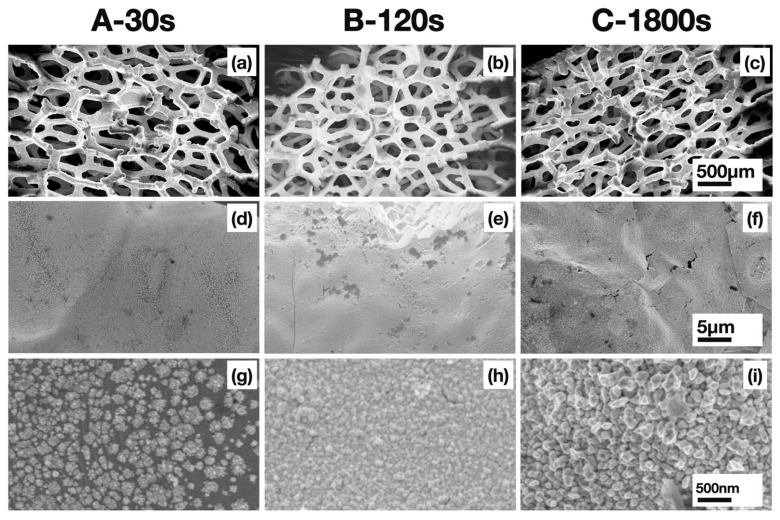
SEM images at different magnifications of Au nanoparticles deposited on Ni foam, obtained for samples A (**a**,**d**,**g**), B (**b**,**e**,**h**) and C (**c**,**f**,**i**).

Figure 2 shows the SEM images at different magnifications of the samples A, B and C. The different preparation conditions gave rise to different morphologies. After 30 s with IPA and stirring (sample A) the coverage of the substrate surface was not uniform, leaving areas with exposed nickel. The Au nanoparticles were also analyzed by high resolution transmission electron microscopy, as shown in Figure S2. By increasing the deposition time up to 120 s (sample B), complete surface coverage was achieved. For sample C, deposited for a further 1800 s without IPA and stirring, after the same preparation method as sample B, the increased time did not result in a relevant increase in gold thickness but, rather, in higher roughness, as also confirmed by the cross section SEM images showing a 100 nm and a 110 nm thick gold film for samples B and C, respectively (Figure S3). The film thickness was converted into loading, by adopting a procedure previously reported [37]. The measured Au loading in sample B and C is 0.08 and 0.09 mg cm^−2^, respectively.

Moreover, the inside and outside of the nickel foam after deposition shared the same morphology. This is because stirring and isopropyl alcohol promote the diffusion of AuCl_4_^−^ ions within the nickel foam, ensuring good deposition conformity, as shown in the SEM images in Figure S4. In particular, for sample C it was possible to achieve a robust gold film, even in the inner part of the Ni foam.

### 3.2. Electrochemical Characterization

**Figure 3:**
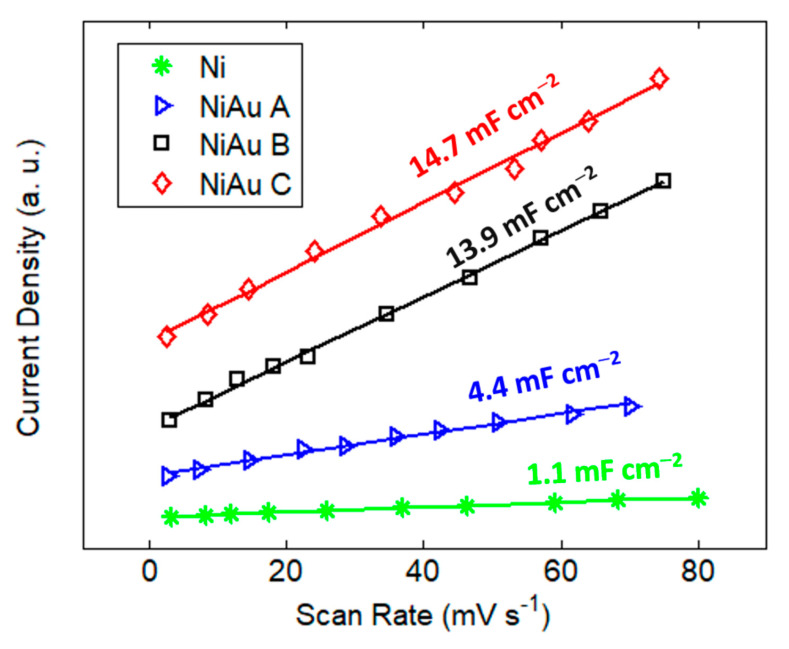
Double layer capacitance calculated from cyclic voltammetry in 0.1 M Na_2_SO_4_ for the Ni foam electrodes loaded with Au catalyst.

For the electrochemical evaluation of the catalysts, a series of CV scans was acquired at different rates in a non-Faradaic region. From these scans, we determined the specific capacitance, which is directly related to the electrochemical active surface area (EASA), by analyzing the slope of the linear regression between the current density differences (cathodic and anodic) in the middle of the potential window of CV curves versus the scan rates. The data shown in Figure 3 reveal that the electrode C had the highest specific capacitance (14.7 mF cm^−2^). This result suggests that the higher roughness of sample C was effective in improving the active sites. The lowest catalytically active surface area was associated with electrode A, exhibiting the lowest capacitance (4.4 mF cm^−2^), due to the non-optimal gold coating of the nickel foam, but this was still higher than that of the bare Ni foam.

These results were also confirmed by EIS (Figure S5 and Table S1), with the highest double layer capacitance obtained for sample C, and a decrease in the charge transfer resistance as the amount of gold increased.

**Figure 4:**
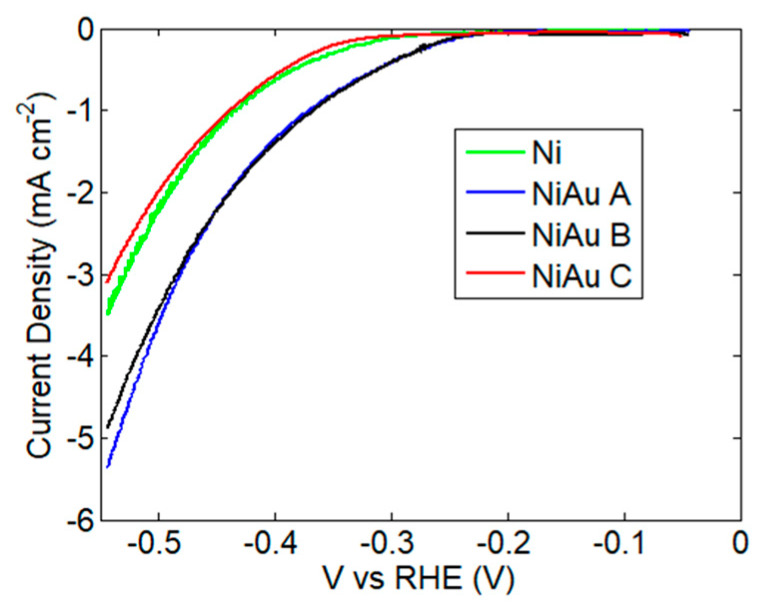
Linear sweep voltammetry in 0.1 M Na_2_SO_4_ with a scan rate of 0.4 mV s^−1^, on bare Ni foam and for electrodes loaded with Au, adopting different deposition conditions.

Figure 4 shows the comparison between the linear sweep voltammetry (LSV), obtained with catalysts prepared in different conditions. The comparison between LSV curves acquired under Ar and N_2_ flux is shown in Figure S6. A higher current was obtained under N_2_ flux, in a region between −0.1 V and −0.3 V vs. RHE. For a potential more negative than −0.3 V vs. RHE, the hydrogen evolution reaction was dominant. The overpotential for HER, taken at 3 mA cm^−2^, was 545 mV for sample C, with a slight decrease to 528 mV for the bare Ni foam. Samples A and B exhibited higher activity in the hydrogen evolution reaction, with an overpotential of 487 mV and 482 mV, respectively.

Since the competition with HER is one of the key issues for ammonia synthesis in water, we studied the electrocatalytic activity for HER using the Tafel plot method. On a single electrode, for a cathodic reaction, the Tafel equation can be stated as:

η = A log10 (I/I0)(3)

where η is the overpotential, A is the Tafel slope, I is the current density and I0 is the current at equilibrium.

In general, the Tafel analysis utilizes the sensitivity of the electric current response to the applied potential (Tafel slope) to obtain information about the rate determining steps. However, the Tafel slope depends not only on the reaction mechanism and on the intrinsic activity of the material toward the process, but also on its three-dimensional structure, including porosity and roughness [45]. In the case of multi-component electrodes, Tafel polarization plots are difficult to analyze, because the same reaction can occur at different rates at different components and can also have different reaction mechanisms. For these reasons, since HER may occur simultaneously on Au and Ni, in this paper, the Tafel plots are used to perform comparative analysis of the activity of Ni foam and of Ni foam functionalized with Au nanostructures with a different morphology and surface coating. The HER is generally described as having three steps: the Volmer step, the Heyrovsky step and the Tafel step. In the Volmer step, a water molecule attacks the catalyst surface and then dissociates, leaving an adsorbed hydrogen atom and a hydroxyl group. In the Heyrovsky step, another water molecule attacks the adsorbed hydrogen and forms a hydrogen molecule and one more hydroxyl group. The H_2_ desorption occurs in both the Tafel and Heyrovsky steps. As is widely accepted, Tafel slopes of 120, 40 and 30 mV dec^−1^ have been observed for the Volmer, Heyrovsky and Tafel determining rate steps, respectively [46].

**Figure 5:**
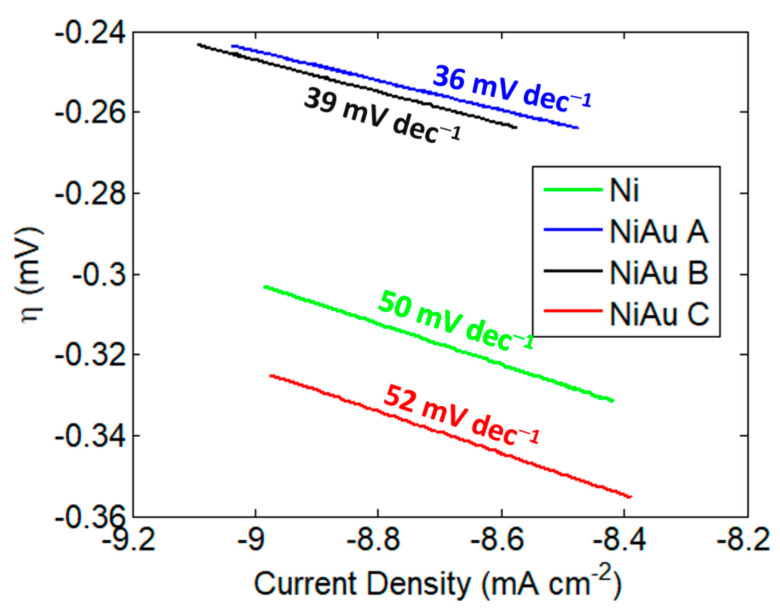
Tafel plot acquired in a quasi-steady-state condition by applying a sweeping rate of 0.4 mV s^−1^ in 0.1 M Na_2_SO_4_.

Figure 5 shows the Tafel slopes obtained for the studied electrodes. The bare Ni foam exhibited a slope of 50 mV dec^−1^. This value decreased to 36 and 40 mV dec^−1^ in samples A and B, respectively. Generally, according to the volcano plot for HER, the Au electrodes exhibit low binding energy and high instability in the H-Au bond (Volmer step), resulting in high overpotential for the HER. Previous DFT calculations [47] have revealed that the binding energy of H-Au on AuNi was more significant than that on Au, because the Au atom adjacent to the Ni atom promotes the Volmer step and enhances HER activity, as confirmed by our experimental decrease in the Tafel plot, observed for samples A and B. In sample C, instead, the Tafel slope is higher and comparable to the bare Ni foam, indicating that, in this sample, the rate for hydrogen evolution is lower. On the basis of this observation, sample C is expected to be the most suitable for nitrogen reduction.

### 3.3. Electrochemical Synthesis of Ammonia

**Figure 6:**
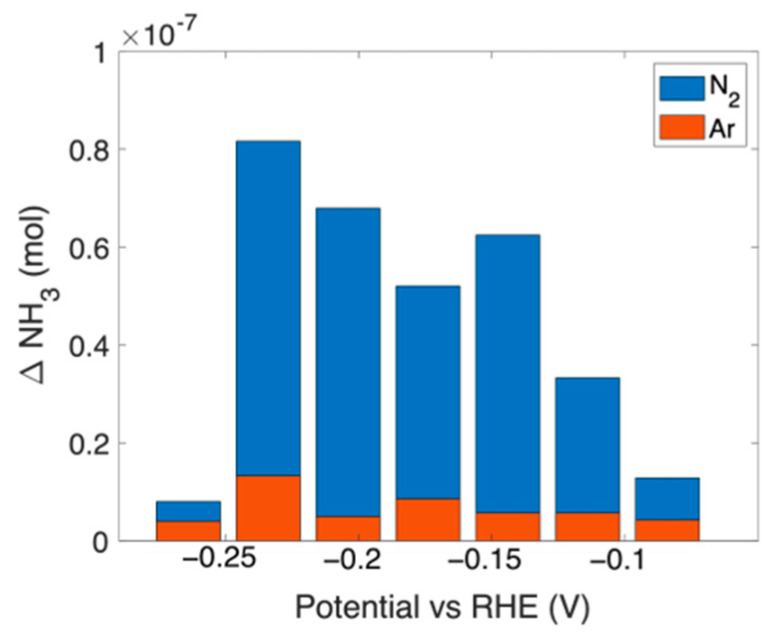
Ammonia moles measured for sample C after chronoamperometry for 2400 s under N_2_ or Ar flux. The amount of moles was subtracted from those measured before the chronoamperometry (open circuit).

Figure 6 shows the net amount of ammonia (ΔNH_3_) produced by 1 cm^2^ of sample C during chronoamperometry at different potentials for 2400 s under N_2_ or Ar flux. Each value reported in Figure 6 is the difference between the ammonia measured after chronoamperometry (CA) and before, at open circuit (Open2). The values measured after each step (Open and CA) at different potentials are shown in Figure S7. Figure S8 shows the UV–Vis spectra after chronoamperometry at −0.23 V vs. RHE, under N_2_ or Ar flux, compared with open and electrolyte.

From Figure 6 it can be seen that a low amount of ammonia (less than 1 × 10^−8^ moles) is produced, even by performing the experiment under Ar flux. This production can be ascribed to the reduction, to ammonia, of NO_x_^−^ present in the deionized water (see Figure S9).

Therefore, the amount of ammonia *produced* by the catalyst was calculated as:

NH_3_ (*produced*) = ΔNH_3_ (N_2_) − ΔNH_3_ (Ar) (4)

**Figure 7:**
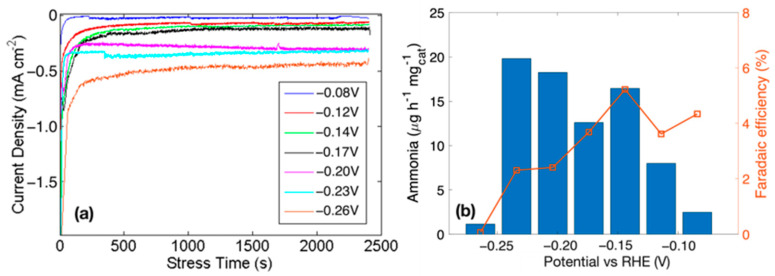
NRR measurements for Ni foam Au 30 min electrode in 0.1 M Na_2_SO_4_: (**a**) chronoamperometry results under N_2_ flux and (**b**) NH_3_ production rate and Faradaic efficiency at different potentials. The ammonia rate and efficiencies are Ar-subtracted (see Figure 6).

Figure 7a shows the chronoamperometry at different potentials. Figure 7b reports the ammonia production rate on the left axis and the faradaic efficiency on the right axis. The maximum NH_3_ yield rate is at −0.23 V vs. RHE with 20 µg h^−1^ mg^−1^_cat_, and the highest Faradaic efficiency of 5.22% is obtained at −0.14 V vs. RHE. The decrease in efficiency observed at more negative potential, is due to the competition of the HER with the NRR.

In order to verify the better performance for sample C, characterized by a higher active area and a higher Tafel slope for HER, we have performed the same characterization under NRR, using sample B. The results are shown in Figure S10. The highest rate and efficiency values are comparable, but in the case of sample B, the rate drastically decreases for potentials more negative than −0.15 V vs. RHE, confirming that the competition with HER plays a relevant role, therefore confirming the validity of the approach proposed for the screening of catalysts as more promising for NRR.

## 4. Conclusions

In summary, this study demonstrated successful gold electroless deposition, based on spontaneous galvanic displacement, on porous Nickel foam.

The deposition time significantly impacted the catalyst morphology and coverage. SEM analysis revealed that longer deposition times resulted in a good deposition uniformity and in a higher roughness of the gold film, without a substantial increase in thickness. y. The electrochemical characterization indicates that the electrode with the highest roughness exhibits the highest active surface area. Tafel slope analyses indicated a poorer performance for HER, leading to a better performance for NRR.

## Data Availability

The data of this study are available from the corresponding author upon reasonable request.

## Supplementary Materials

The following supporting information can be downloaded at: https://www.mdpi.com/article/10.3390/nano13212850/s1, Figure S1: (a) UV-Vis absorbance spectra for the indophenol method with various ammonia concentrations and (b) the corresponding calibration curve; Figure S2: High Resolution Transmission Electron Micrograph of Au nanoparticles deposited for 30 s in sample A; Figure S3: Cross Section SEM images of Au deposited on Ni Foam obtained for samples B and C. The cross section has been prepared by cutting the foam and acquiring the micrographs in a region with the delaminated Au film. The thickness is 100 nm for sample B and 110 nm for sample C; Figure S4: SEM images of the outer (out) and inner (inn) regions of Ni Foam, as indicated in the inset and after the gold deposition for sample A and for sample B; Figure S5: Electrochemical impedance spectra of gold electrodes acquired in 0.1 M Na_2_SO_4_, at 0 V bias with a small signal 50 mV in amplitude; Figure S6: Linear Sweep Voltammetry obtained with sample A, B and C, respectively, scanning the potential from +0 to −0.55 V vs. RHE under Ar (black line) and N2 (red line) flux in 0.1 M Na_2_SO_4_; Figure S7: Ammonia moles measured for sample C after (labeled as CA) and before (Open2) chronoamperometry for 2400 s under N_2_ and Ar flux; Figure S8: UV-Vis absorbance spectra for the electrolyte, before (Open 1, after 10 min and Open2, after 40 min respectively under gas flow and with no voltage applied) and after the chronoamperometry experiments in argon and nitrogen flux at −0.23 V vs. RHE [48]; Figure S9: NOx calibration by HCl with UV spectrometry absorbance spectra and calibration curve; Figure S10: Ammonia moles measured for sample B after (labeled as CA) and before (Open2) chronoamperometry for 2400 s under N_2_ flux and NH_3_ production rate and Faradaic Efficiency at different potentials. Table S1. Fitted values for double layer capacity and charge transfer resistance.
