# Supplementary material for: Ultra-Low Loading of Gold on Nickel Foam for Nitrogen Electrochemistry
Source: Nanomaterials (Basel). 2023 Oct 27;13(21):2850. doi: 10.3390/nano13212850 (PMC10647533; doi:10.3390/nano13212850)
Supplement: Supplementary file 1 [file nanomaterials-13-02850-s001.zip › nanomaterials-2665091-supplementary.pdf]

Supplementary info for:

Ultra low loading of gold on nickel foam for nitrogen electrochemistry

Giuseppe Tranchida [1-2], Rachela G. Milazzo* [2], Marco Leonardi [1-2], Silvia Scalese[2], Roberta A. Farina [1-2], Salvatore Lombardo[2] and Stefania M.S. Privitera[2]

1) Dipartimento di Scienze Chimiche, Università degli Studi di Catania, Viale Andrea Doria, 6, 95125 Catania, Italia;
2) Consiglio Nazionale delle Ricerche, Istituto per la Microelettronica e i Microsistemi (CNR-IMM), Strada VIII, 5, 95121 Catania, Italia

Correspondence: gabriella.milazzo@imm.cnr.it

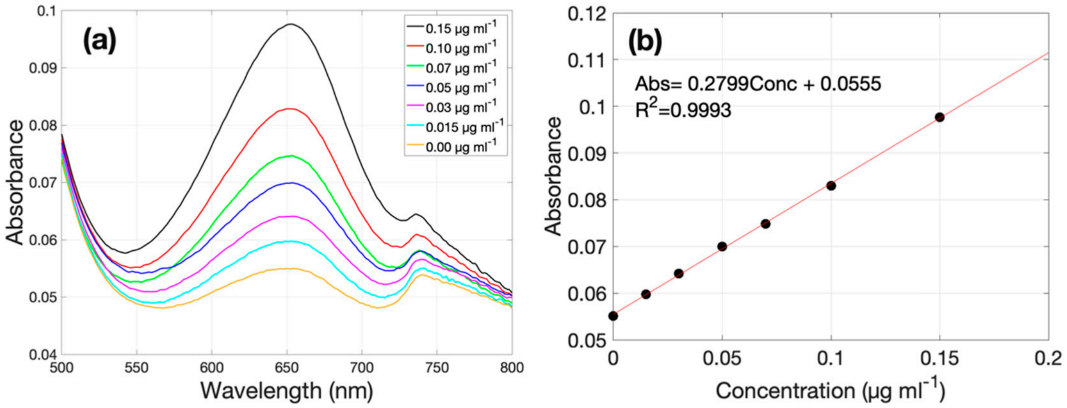

Figure S1. (*a*) UV-Vis absorbance spectra for the indophenol method with various ammonia concentrations and (*b*) the corresponding calibration curve.

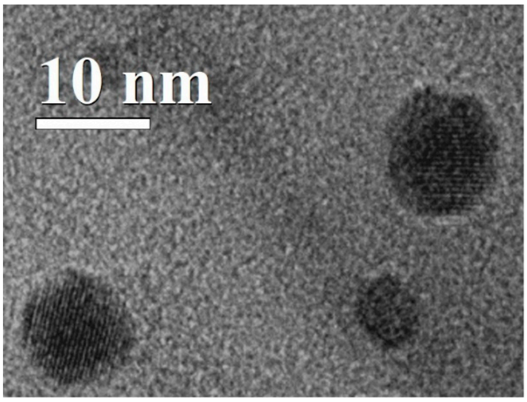

Figure S2: High Resolution Transmission Electron Micrograph of Au nanoparticles deposited for 30s in sample A.

The analysis has been performed with a JEOL 2010F Microscope operating at 200kV.

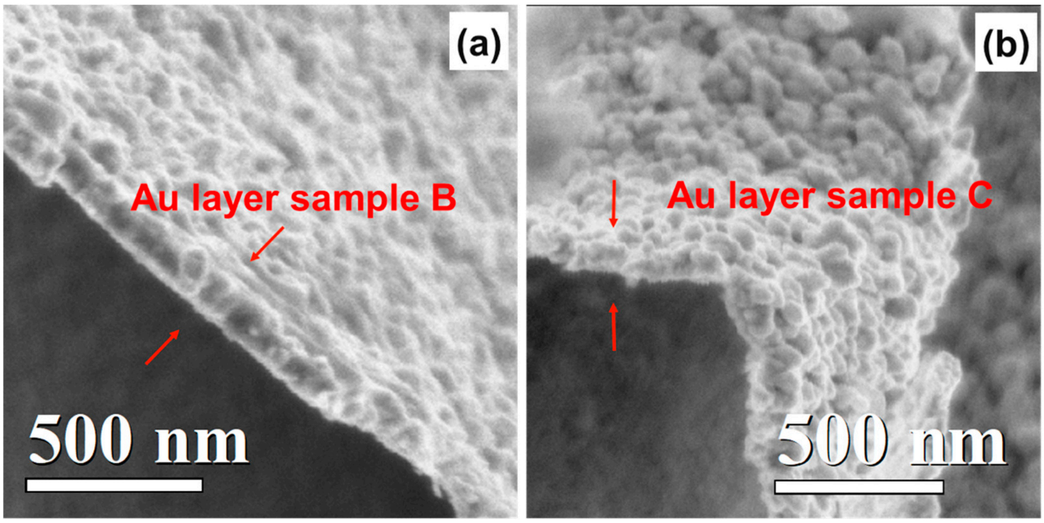

Figure S3. Cross Section SEM images of Au deposited on Ni Foam obtained for samples B and C. The cross section has been prepared by cutting the foam and acquiring the micrographs in a region with the delaminated Au film. The thickness is 100nm for sample B and 110nm for sample C.

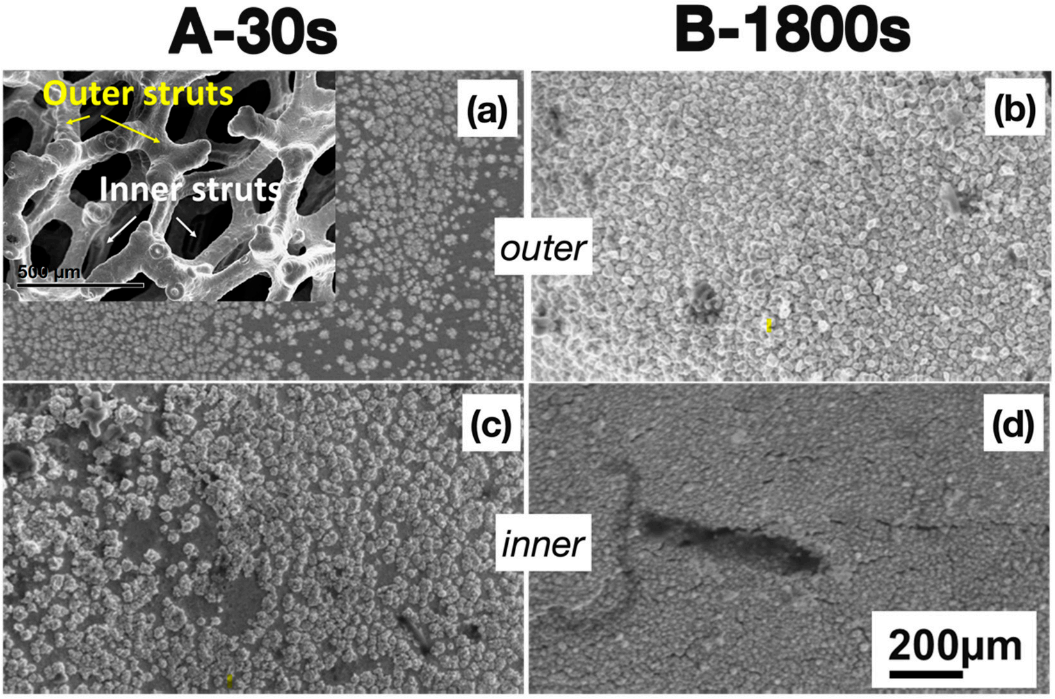

Figure S4. SEM images of the outer (out) and inner (inn) regions of Ni Foam, as indicated in the inset and after the gold deposition for sample A (**a**,**c**) and for sample B (**b**,**d**).

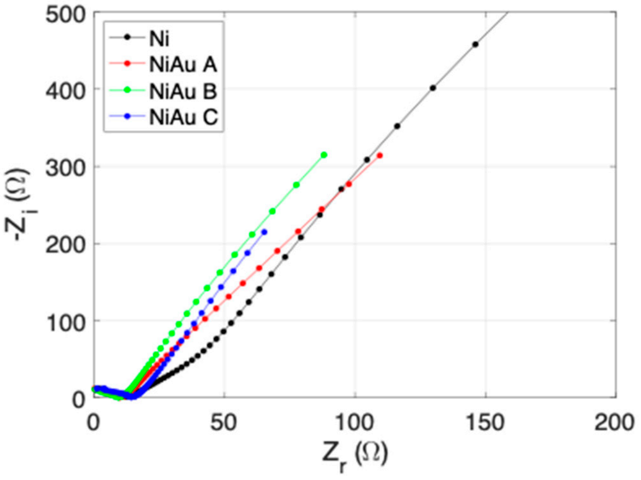

Figure S5. Electrochemical impedance spectra of gold electrodes acquired in 0.1M Na_2SO_4, at 0V bias with a small signal 50mV in amplitude. The results are shown in Table S1.

Sample	C double Layer (F)	R charge Transfer (Ω)
Ni Foam	3.67E-04	3370
Ni Foam Au 30 s	4.36E-04	813
Ni Foam Au 120 s	5.32E-04	1630
Ni Foam Au 1800 s	8.40E-04	1610

Table S1. Fitted values for double layer capacity and charge transfer resistance.

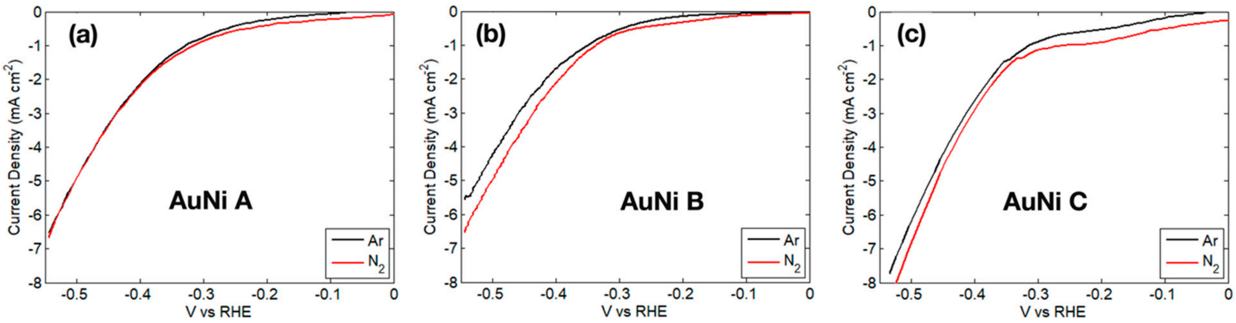

Figure S6. Linear Sweep Voltammetry obtained with sample A, B and C, respectively, scanning the potential from +0 to -0.55 V vs RHE under Ar (black line) and N2 (red line) flux in 0.1M Na_2SO_4.

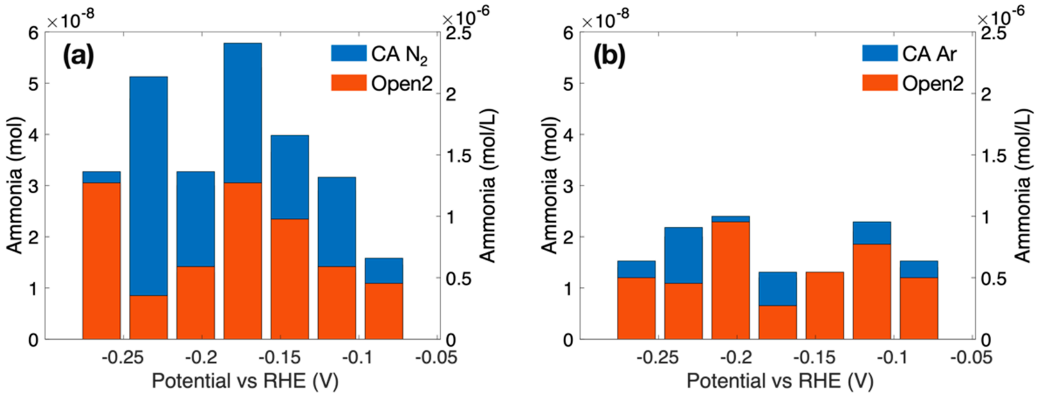

Figure S7. Ammonia moles measured for sample C after (labeled as CA) and before (Open2) chronoamperometry for 2400s under (**a**) N_2 and (**b**) Ar flux.

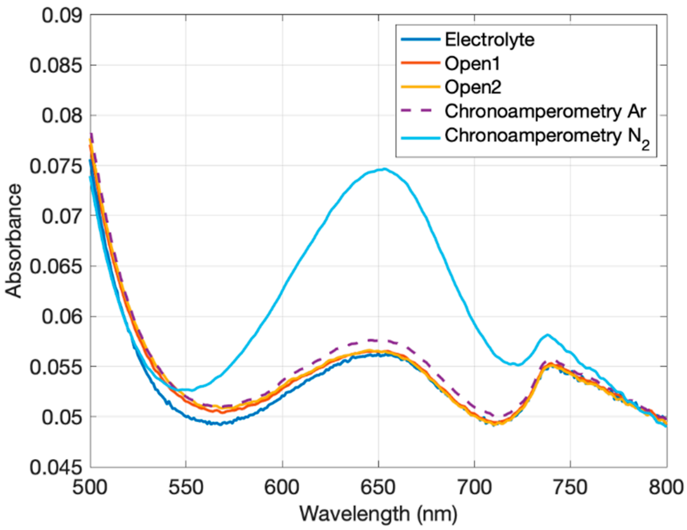

Figure S8. UV-Vis absorbance spectra for the electrolyte, before (Open 1, after 10min and Open2, after 40min respectively under gas flow and with no voltage applied) and after the chronoamperometry experiments in argon and nitrogen flux at -0.23 V vs RHE.

Nitrate analyses

We evaluated the NOx by UV spectrometry at 215nm, with HCl method [48]. The NO_x^- amount measured in the deionized water was calculated by using the standard addition method, obtaining 1.4 x 10^{-6} M. In 22ml we therefore expect about 3 x 10^{-8} moles. To evaluate possible NO_x^- contaminations introduced by gas fluxing we adopted MilliQ water to ensure higher sensitivity. After 40 min the increase in the NO_x^- concentration was no higher than 1 x 10^{-6} M. After 80 min fluxing Ar or N_2, 4.4 x 10^{-8} moles and 5.5 x 10^{-9} moles were introduced, respectively.

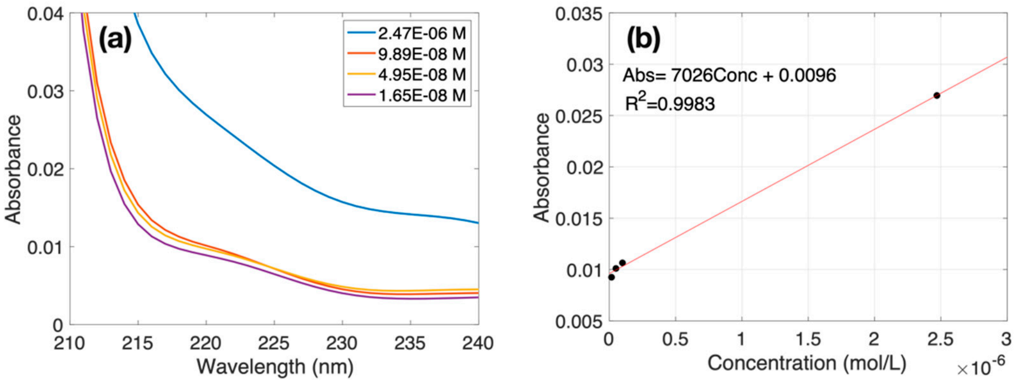

Figure S9. NOx calibration by HCl with UV spectrometry (**a**) absorbance spectra and (**b**) calibration curve.

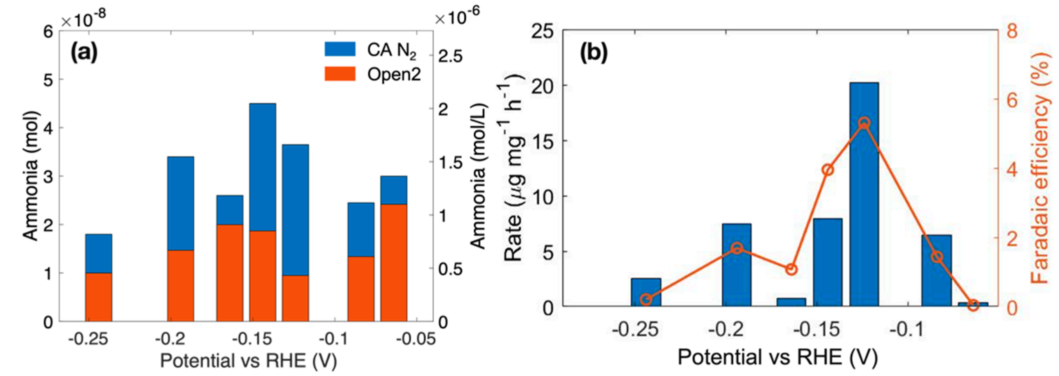

Figure S10. (**a**) Ammonia moles measured for sample B after (labeled as CA) and before (Open2) chronoamperometry for 2400s under N_2 flux and (**b**) NH_3 production rate and Faradaic Efficiency at different potentials.
